# Development of a novel multiplex DNA microarray for *Fusarium graminearum *and analysis of azole fungicide responses

**DOI:** 10.1186/1471-2164-12-52

**Published:** 2011-01-21

**Authors:** Rayko Becher, Fabian Weihmann, Holger B Deising, Stefan GR Wirsel

**Affiliations:** 1 Institut für Agrar- und Ernährungswissenschaften, Naturwissenschaftliche Fakultät III, Martin-Luther-Universität Halle-Wittenberg, Betty-Heimann-Str. 3, D-06120 Halle (Saale), Germany; 2 Interdisziplinäres Zentrum für Nutzpflanzenforschung, Martin-Luther-Universität Halle-Wittenberg, Betty-Heimann-Str. 3, D-06120 Halle (Saale), Germany

## Abstract

**Background:**

The toxigenic fungal plant pathogen *Fusarium graminearum *compromises wheat production worldwide. Azole fungicides play a prominent role in controlling this pathogen. Sequencing of its genome stimulated the development of high-throughput technologies to study mechanisms of coping with fungicide stress and adaptation to fungicides at a previously unprecedented precision. DNA-microarrays have been used to analyze genome-wide gene expression patterns and uncovered complex transcriptional responses. A recently developed one-color multiplex array format allowed flexible, effective, and parallel examinations of eight RNA samples.

**Results:**

We took advantage of the 8 × 15 k Agilent format to design, evaluate, and apply a novel microarray covering the whole *F. graminearum *genome to analyze transcriptional responses to azole fungicide treatment. Comparative statistical analysis of expression profiles uncovered 1058 genes that were significantly differentially expressed after azole-treatment. Quantitative RT-PCR analysis for 31 selected genes indicated high conformity to results from the microarray hybridization. Among the 596 genes with significantly increased transcript levels, analyses using GeneOntology and FunCat annotations detected the ergosterol-biosynthesis pathway genes as the category most significantly responding, confirming the mode-of-action of azole fungicides. *Cyp51A*, which is one of the three *F. graminearum *paralogs of *Cyp51 *encoding the target of azoles, was the most consistently differentially expressed gene of the entire study. A molecular phylogeny analyzing the relationships of the three CYP51 proteins in the context of 38 fungal genomes belonging to the Pezizomycotina indicated that CYP51C (FGSG_11024) groups with a new clade of CYP51 proteins. The transcriptional profiles for genes encoding ABC transporters and transcription factors suggested several involved in mechanisms alleviating the impact of the fungicide. Comparative analyses with published microarray experiments obtained from two different nutritional stress conditions identified subsets of genes responding to different types of stress. Some of the genes that responded only to tebuconazole treatment appeared to be unique to the *F. graminearum *genome.

**Conclusions:**

The novel *F. graminearum *8 × 15 k microarray is a reliable and efficient high-throughput tool for genome-wide expression profiling experiments in fungicide research, and beyond, as shown by our data obtained for azole responses. The array data contribute to understanding mechanisms of fungicide resistance and allow identifying fungicide targets.

## Background

The ascomycete *Fusarium graminearum *Schwabe (teleomorph *Gibberella zeae *(Schweinitz) Petch) is an important member of the causal agents of the Fusarium Head Blight (FHB) complex in cereals, a disease of global concern in wheat and barley [1]. Typically, spores dispersed by raindrops or wind infect flowering spikelets during humid weather at anthesis which may lead to serious FHB under post-flowering moisture conditions [1,2]. Like in FHB, several *Fusarium *species including *F. graminearum *are responsible for a disease on basal stem tissue of cereals, including maize, called crown rot. Beside considerable yield losses, drastic quality losses may result from contamination of grains with mycotoxins produced by *F. graminearum*. Like other members of the genus, *F. graminearum *produces a mixture of several mycotoxins, including deoxynivalenol (DON) that cause severe threats to human and animal health [3]. National governments have established strict regulations for tolerable mycotoxins levels in cereal products.

Azole fungicides are indispensable for the control of FHB. These fungicides inhibit the cytochrome P450 sterol 14α-demethylase (CYP51, syn. ERG11), an enzyme that is essential for ergosterol biosynthesis, leading to disturbance of fungal membrane integrity. The current limitation of alternative fungicides to control effectively FHB has resulted in increased application of azoles. As a result, strains exhibiting increased resistance to this fungicide class were recovered from *F. graminearum *field populations [4,5]. Declining efficacies of azoles have also been reported in other fungal cereal pathogens, e.g. *Septoria tritici *and *Blumeria graminis *[6,7]. The molecular basis of fungicide resistance, however, is unclear in many cases. Fungicides currently used in agriculture only address a limited number of molecular targets, most of which are enzymes of the ergosterol biosynthetic pathway. Thus, the discovery of novel fungicides with novel modes-of-action would represent a break-through in chemical plant protection. Genome-wide transcriptome analysis may help suggesting mechanisms of drug resistance [8].

The importance of *F. graminearum *for agronomy but also for basic research stimulated efforts to sequence its genome [9]. Recently, the third improved annotation of the genome (FG3) that lists 13,332 genes has become available. Gene calls from the first genome annotation by the Broad Institute (Cambridge, MA, USA) and from an annotation by the MIPS Institute (München, Germany) were combined to develop a DNA microarray using the Affymetrix GeneChip format [10]. While the Affymetrix photolithographical method allowed synthesis of short oligonucleotides (25 mers), the more recently developed ink jet procedure of Agilent synthesizes longer 60 mer oligonucleotides *in situ* which increased the detection sensitivity [11]. In addition, for Agilent several multiplex array formats are available. The Agilent 8 × 15 k format holds eight independent microarrays on a single slide, each carrying up to 15,000 spotted oligonucleotides, which is sufficient to cover most fungal genomes with at least one probe per gene. As demonstrated previously, the high specificity and affinity of long oligomer probes allow representing genes on microarrays by a single probe [12].

In this study, we developed a novel microarray designed for the 8 × 15 k Agilent multiplex format. To validate its reliability for expression profiling experiments, this array was employed to determine the genome-wide transcriptional response of *F. graminearum *to treatment with the sterol biosynthesis inhibitor tebuconazole. The application of recent microarray statistics and functional gene enrichment approaches uncovered the most significant responses for several sterol biosynthesis pathway genes. In addition, amongst the many genes encoding ABC transporters and transcription factors, some were identified that showed significantly altered transcript levels that were only observed in our tebuconazole study but not in a previously published microarray experiment that examined starvation stress conditions.

## Methods

### Fungal cultivation

In this study, we used *Fusarium graminearum *strain NRRL 31084 (syn. PH1). To warrant high reproducibility, conidial stock suspensions (10^6 ^conidia/ml) were stored in 20% (v/v) glycerol at -80°C. Fungal cultivation and preparation of conidia suspension were performed as described before [13].

To prepare RNA and genomic DNA used for the microarray preselection experiment (see below), 10^6 ^conidia were inoculated into three 300 ml flasks containing 100 ml complete medium (CM) [14] and cultivated at 23°C with shaking at 150 rpm. Mycelia were harvested after 24 h, washed with sterile distilled water and re-inoculated into 100 ml of CM, minimal medium (MM) [14] lacking a nitrogen source (MMN) or MM without a carbon source (MMC). After 12 h of cultivation at 23°C with shaking at 150 rpm, the mycelia were harvested, frozen in liquid nitrogen, and kept at -80°C until preparation of nucleic acids. For the preparation of RNA used for microarray experiments investigating azole responses, 10^6 ^conidia were inoculated into six 300-ml-flasks containing 100 ml CM and incubated as above. After 24 h, three of the cultures were adjusted to 5 mg/l tebuconazole. Incubation of all cultures continued for further 12 h, and cultures were harvested as described above.

### Nucleic acid isolation

Total RNA was isolated from mycelia grown *in vitro *with the RNeasy Plant Mini Kit (Qiagen, Hilden, Germany) following the manufacturer's protocol. The optional on-column DNase digestion step was performed using RNase-free DNase (Qiagen). Genomic DNA was isolated from mycelium cultured in CM as described [15]. Quantity and purity of nucleic acid preparations were determined using a NanoDrop ND-1000 UV-VIS spectrophotometer (Thermo Scientific, Wilmington, USA). RNA quality was assessed with a BioAnalyzer 2100 (Agilent Technologies, Böblingen, Germany).

### Microarray development

The microarray was designed in collaboration with an Agilent-certified service provider (imaGenes, Berlin, Germany) that applied an in-house developed approach for selection of the best performing probes for each gene (http://www.imagenes-bio.de/services/microarray/pss/mechanism). Using the annotation FG3, for each gene model, several 60 mer oligonucleotides were selected by the software eArray (https://earray.chem.agilent.com/earray/) and were then filtered by proprietary software (imaGenes). In addition to the 13,332 genes of the FG3 annotation, available cDNA sequences of the TRI (= trichothecene biosynthesis) gene cluster from nivalenol (NIV) producing strains of *F. graminearum*, i.e. strains H88-1 (accessions: AAK53574 to AAK53581, AAM22490) and NRRL 13383 (accessions: AAM 48747 to AAM48754) were chosen for oligonucleotide selection. The inclusion of these cDNA sequences was considered useful as genes of the TRI cluster of NIV-producing strains show several differences that might not reliably allow quantitative detection with oligonucleotides derived from DON-type PH1 sequences. Throughout this study, data from these additional cDNA-based probes were excluded from analyses.

Thereafter, up to nine oligonucleotides per gene were assembled on a pre-selection array using the Agilent 2 × 105 k format and hybridized against cyanine 3 (Cy3)-labeled genomic DNA and cRNA obtained from pooled total RNA derived from three cultures grown in CM, MMN, and MMC. Based on the RNA hybridization results, for each gene the relative ratio of probes was determined that yielded significant signal intensities. In case that this ratio was 60% higher than the background, a gene was considered expressed. For these genes, final oligonucleotide selection relied on the results from RNA hybridization, whereas for genes considered non-expressed hybridization results from genomic DNA were used. The final array design applied a proprietary algorithm (imaGenes) that used different criteria for expressed and non-expressed genes. The optimal oligonucleotides finally assembled in the 8 × 15 k Agilent microarray format for analyzing transcriptomic responses of *F. graminearum *is publicly available (Additional file 1). Microarrays can be ordered at Agilent using design ID 021301. Since this format is highly flexible in production it can easily be updated if future annotations should modify some gene models.

### Microarray sample preparation and hybridization

For sample preparation and array processing the Agilent protocol "One-color microarray-based gene expression analysis (Quick Amp Labeling)" was used (http://www.chem.agilent.com). Briefly, the recommended volume of control RNAs (Agilent One-Color RNA Spike-In Kit) was added to 200 ng of total RNA. Thereafter, Cy3-labeled cRNA was produced, using the Agilent Quick Amp Kit (one-color) according to the manufacturer's protocol. Labeled cRNA was purified with the RNeasy mini kit (Qiagen). Yield and Cy3 incorporation rates were analyzed using a NanoDrop ND-1000 UV-VIS spectrophotometer. In addition, size distribution of cRNAs was assessed by a BioAnalyzer 2100. Genomic DNA was Cy3-labeled using the Genomic DNA Enzymatic Labeling Kit (Agilent) according to the manufacturer's recommendations.

Hybridization of the 105 k pre-selection arrays was performed with either 5 μg or 1.5 μg of the Cy3-labeled gDNA or cRNA derived from a pooled RNA sample as described above. For hybridizations using the 15 k arrays, 600 ng of Cy3-labeled cRNA was used. Hybridization was performed with eight cRNA samples. Six cRNA samples derived from three independent untreated and three independent azole-treated liquid cultures (see above). The remaining two samples represented additional technically repeated hybridizations that used the labeled cRNAs from one of the untreated cultures. Hybridization was set up in a Microarray Hybridization Chamber (Agilent) and was incubated in a hybridization oven (Agilent) at 65°C and 10 rpm for 17 h. After performing the suggested washing steps, slides were scanned by a microarray scanner (Agilent) at 5 μm resolution.

### Normalization and statistical analysis of microarray data

The microarray scan data were analyzed by Agilent's Feature Extraction software, which estimates the background level and gives a feature-specific background cut-off level per chip. The background-corrected raw signals were converted to gene signals by a median polish algorithm as implemented in the RMA (robust multi-array average) algorithm [16]. Gene signals were normalized between experiments by quantile normalization [17].

Relying on the normalized gene signals, the average log_2 _fold-changes between both experimental groups (tebuconazole-treated vs. untreated) were assessed for each gene. We applied the optimal discovery procedure (ODP) to identify significant changes in relative transcript levels [18]. This combinatorial statistical approach is available in the open-source software package EDGE version 1.1.291 [19]. The identification of differentially expressed genes includes two steps. First, ODP statistics is applied for each gene, which results in a ranking of the genes from most to least differentially expressed. In a second step, statistical significance is estimated by using the false discovery rate (FDR) methodology [20] which results in the assignment of a *q*-value for each gene. The *q*-value gives the expected rate of false positives amongst those genes that were assigned as differentially expressed.

Datasets of processed signal intensities from biological and technical replicates were evaluated with Pearson's coefficient of correlation (R) using MS Excel 2007 (Microsoft, Redmond, USA).

### Bioinformatics

#### Functional enrichment analyses

Gene sets were examined for significant enrichment of functional categories using two annotation schemes, i.e. "The Functional Catalogue" (FunCat) [21] and "GeneOntology" (GO) [22]. The FunCat annotation of the *F. graminearum *genome was provided by the MIPS *Fusarium graminearum *database (FGDB) (http://mips.helmholtz-muenchen.de/genre/proj/FGDB/). We performed the GeneOntology annotation of the *F. graminearum *genome by subjecting all 13,332 proteins of the FG3 annotation to Blast2GO (version 2.3.6) analysis (http://www.blast2go.org). This software extracts GO terms characterizing the putative function of the proteins relying on ontologies mapped to homologous proteins identified by BLAST [23]. BlastP parameters that differed from the default were set as follows: "Number of Blast Hits" at 30, "Blast ExpectValue" at 1.0E-10. Furthermore, the "Try SIMAP first" option was chosen. The GO annotation was performed with default settings except for the "Annotation Cutoff" value that was set to 30. Thereby, Blast2GO could assign 8463 proteins to at least one of the general GO categories of biological process, molecular function, and cellular component. In analogy to FunCat enrichment analysis at the FGDB server, assessment of statistically significant GO term enrichment utilized the hypergeometric distribution function as implemented in MS Excel. The resulting *p *values from both, the FunCat and the GO enrichment analyses, were corrected for multiple testing by applying the Benjamini-Hochberg FDR procedure [24]. The cutoff for FDR-corrected *p *values was set to 0.05.

#### Identification and assignment of putative homologs

Microarray data analyses were focused, amongst others, on genes of the ergosterol biosynthesis pathway. For this purpose, at first sequences of *Saccharomyces cerevisiae *proteins known to be involved in ergosterol biosynthesis [25] were retrieved from the *Saccharomyces *Genome Database (SGD) (http://www.yeastgenome.org/). These sequences were used as queries in the BlastP search option implemented at the FGDB server (see above) to retrieve putative homologs from the *F. graminearum *proteome (see Additional file 2).

We examined the phylogenetic relationships of the CYP51/ERG11 proteins from *F. graminearum *by comparison to the corresponding proteins of other fungi (see below). To identify such proteins in 38 currently annotated proteomes of the Pezizomycotina we used the ERG11 sequence from *Saccharomyces cerevisiae *as the query in BlastP searches at NCBI Genomic Blast (http://www.ncbi.nlm.nih.gov/sutils/genom_table.cgi), at the Joint Genome Institute database (http://www.jgi.doe.gov/genome-projects), or at the NITE Institute database (http://www.bio.nite.go.jp/dogan).

Furthermore, we assessed the phylogenetic relationships between all putative ATP-binding cassette (ABC) transporters from *F. graminearum*. The identification of such proteins relied on the FunCat and GO annotations described above. FGDB provided 78 genes annotated in the FunCat category 'ABC transporters' (20.03.25). Manual revision appeared to be necessary since this list also contained MFS (= major facilitator superfamily) transporters and other non-ABC proteins. Beyond those, we excluded four additional proteins that lacked any transmembrane domains, which were identified by the TMHMM software (http://www.cbs.dtu.dk/services/TMHMM/), resulting in a set of 50 putative ABC transporters. In a second analysis using the GO annotations, we applied the GO term 'ATPase activity, coupled to transmembrane movement of substances' (GO:042626). This recovered four additional proteins that were not found by FunCat and which were included in the molecular phylogeny. To assign all identified ABC transporters to one of the known subfamilies [26,27] we analyzed the distribution of predicted transmembrane and ATP binding domains by the TMHMM (see above) and the Conserved Domain Search (CDS) softwares (http://www.ncbi.nlm.nih.gov/Structure/cdd/cdd.shtml). In addition, BlastP searches using ABC transporters from *S. cerevisiae *and *C. albicans *as queries assisted in the classification of the corresponding *F. graminearum *proteins.

Predicted putative *F. graminearum *transcription factors were obtained from the Fungal Transcription Factor Database (FTFD) (http://ftfd.snu.ac.kr/). Transcription factors whose transcript levels differed significantly between the control and the fungicide treatment in the microarray experiments were used as queries in BlastP searches against the proteomes of *S. cerevisiae *(at SGD; see above) and *Candida albicans *(CGD; http://www.candidagenome.org). Vice versa, best hits were used for BlastP searches against the *F. graminearum *proteome.

#### Phylogenetic analyses

For phylogenetic analyses, we used the software MEGA version 4 [28]. Alignments were created by the implemented ClustalW algorithm, using the substitution BLOSUM weight matrix and the following parameters. For pairwise and multiple alignment the gap opening penalty was set to 10, whereas the gap extension penalties were set to 0.1, and respectively 0.05. Evolutionary history was inferred using the Neighbor-Joining method. Evolutionary distances were computed using the Poisson correction method. Gaps and missing data were eliminated only from pairwise sequence comparisons (Pairwise deletion option). The confidence of the resulting tree was estimated with 1000 bootstrap iterations.

#### Comparative analyses using published microarray data

Microarray data analyzing in *F. graminearum *two nutritional stress conditions, i.e. carbon and nitrogen starvation, were published [10] and deposited at the MIAME-compliant Plant Expression Database (http://www.plexdb.org). The RMA-normalized signal data were assigned to the updated gene names based on the ProbeSet mapping table available at the MIPS FTP sites (ftp://ftpmips.gsf.de/FGDB/) and subjected to the same statistical analyses described above. Genes determined to be differentially expressed in any of the three stress conditions, i.e. tebuconazole treatment, carbon and nitrogen starvation, were assessed for common and specific responses using MS Excel. We performed BlastP searches (http://blast.ncbi.nlm.nih.gov/Blast.cgi) at the database 'Non-redundant protein sequences (nr)' and organism 'fungi (taxid:4751)' to check whether those genes that only responded to tebuconazole were exclusively found in the *F. graminearum *genome.

### Deposition of microarray data

MIAME-compliant information about the 8 × 15 k *F. graminearum *microarray platform and the azole treatment experiment have been deposited in the NCBI Gene Expression Omnibus (GEO) database (http://www.ncbi.nlm.nih.gov/geo) under the accession numbers GPL11158 (platform) and GSE 25114 (experiment).

### Quantitative RT-PCR

qRT-PCR used the Power Sybr Green RNA-to-C_T _1-step Kit (Applied Biosystems, Darmstadt, Germany) and the iCycler model MyiQ Single color (Bio-Rad Laboratories, München, Germany), according to the manufacturer's protocols. Reactions of 20 μl contained 20 ng of the same total RNAs that had previously been used for microarray hybridization. For all reactions, melting curve analyses at the end of the amplification and subsequent electrophoresis on 1.5% agarose gels allowed verifying amplification of a single fragment. Additionally, for each gene assessed, one reaction was purified with the SureClean Kit (Bioline, Luckenwalde, Germany) and sequenced with the BigDye Terminator v1.1 Cycle Sequencing Kit (Applied Biosystems). For each gene analyzed and each RNA preparation used, qRT-PCR was conducted in three technical repeats.

Primers were designed with the software NCBI Primer-BLAST (http://www.ncbi.nlm.nih.gov/tools/primer-blast/) (Table 1). Primers for the reference genes (see below) included a predicted intron, if available, which tested for contamination of RNA preparations with genomic DNA. In case of FGSG_10791, a predicted intron could not be confirmed.

**Table 1:** Primers used for qRT-PCR

**Gene ID **^a^	Forward primer (5'-3')	Reverse primer (5'-3')	Amplicon (bp)
**CYP51**			
FGSG_01000	TCTACACCGTTCTCACTACTCC	GCTTCTCTTGAAGTAATCGC	170
FGSG_04092	CCCTTACACGATCACTACAGAC	CTCTTCGTTCCTTTAGACACAG	186
FGSG_11024	ACTTGGTCTCACCACAGATTC	CATAGATCGGACTTCGTTTC	187
**ABC transporters**			
FGSG_00046	CTACTATCTGGGTGAACCTGTG	GCCACTGTTTTCAGGAGTATC	209
FGSG_02786	CTTCGGCAAGATATGAGCTTC	GCGAGTATGGAACTCGATAGG	161
FGSG_03032	CCTCGCAAGACAACCATACAG	AGAGTGATGTCGCGAATATCG	176
FGSG_03882	GAACTCACGTACTCTCCTCAAC	TCGACATCAACTCTCTCTTGAC	168
FGSG_05076	GTGCTATGCTCCAGCAATACC	CGATCAGAAGAGCGAACTGAG	198
FGSG_06771	TCGTCAGTCCAGAAGTCAAG	TTCGGTTCACAAAGTAGTCC	180
FGSG_06881	AAACCCCGTTGAAACAGAAC	ATCAAACCATAAGCGACGTTC	186
FGSG_07325	CCTCTATGCTTTCCTTCATC	ATACCGTGTCTTCAACTGTCTC	207
FGSG_08308	ACAGGCTCGTCTATTCACAG	GACCCCTTGATAACCTCTAAC	187
FGSG_08309	GTCTCTTTGTATCAGGCTTCC	CATATTCTCTCTCCCACTCATC	199
FGSG_08312	GATCGTCCTCTTCACTATTACC	CTCTCAGCAGTAACCATAGGAC	183
FGSG_08373	CTGTGACGATACCGAGCTGAC	CCTGTATCGAGCCAAATCAAG	120
FGSG_08830	GACTACTTGGCTTCTCTTTCC	CACCATCTTCTTGACTACCAAC	250
FGSG_10995	ATTTATGACCACGCAGTCTGG	CTGCGAAAAGAGGTTCGTTAC	121
FGSG_11028	GACACACTACCGACACAACTG	GACTGAGAAGACGAGAAGAAG	225
FGSG_11988	GATGTAATGCTACCTGGAACAC	GATGAGACCGATTGTGAGAAC	195
**Transcription factors**			
FGSG_00069	GAATCTTCTCCGGCACTCAG	TTCTTCACTTCCTTCCGAGATC	139
FGSG_01293	CCCGATATAGTCGAGCCTAAC	GGTATTTGTTGCTCCGTTGAC	101
FGSG_01341	ATTCTGGCATGGATGATGAAG	CACCAAAGTCACTGGCATATCC	162
FGSG_01669	TTCGACTTCTCACAGCTCAGC	TGCCTGCATGTTGTACTGGTC	120
FGSG_05949	TTCGGCAACCATCACTCCTAG	TGCTGTTCGTTCTCTCGCACT	100
FGSG_06324	ACAGAGTGTCGAACCAGCAAC	GGTTCTCGTGGGATGCTATC	173
FGSG_06810	ATACCCGTCTCATGAACATCG	TCGATAACAGCCTTGGCTATG	127
FGSG_09333	CACAGCTCAACGATGCAATG	CAAGATAAGCAAGGATGCTGTG	101
FGSG_09349	GCTTGATTGCCCCTCTGAGA	GGTTCGCATTCTCCTGGTTC	123
FGSG_10470	AGAGATCACCACGTCCGAAC	TGGTGGGTGTAGATGTGGTTC	114
FGSG_10914	CATCGGGCTCTAGTACGAATC	CCAGAAAGCAGCAGTATGCTC	141
FGSG_11561	ACTCGAAAGCATTGGATCAGC	AGACGGACGAAATCATCGTC	130

^a ^The given gene ID is the entry number of the *F. graminearum *genome annotation FG3 provided by Broad Institute.

Analysis of qRT-PCR data applied the sigmoidal curve-fitting method [29] that calculates the initial fluorescence (R_0_), which relates to the initial amount of the target in a sample. Computation of R_0 _was conducted using the software Sigmaplot version 11 (Systat Software, Erkrath, Germany). As quantification of gene expression requires normalization by stable internal references, a minimum of three reference genes is recommended to derive a combined normalization factor used to adjust the data of the genes of interest [30]. To identify genes with putatively stable expression levels, normalized signals from all hybridizations of the 8 × 15 k microarray data except those from the technical replications were used to calculate a coefficient of variation (CV = SD/mean) for each gene. The least variable genes with CV < 0.05 were examined for a putative housekeeping function, according to the gene descriptions at the FGDB. As a result, FGSG_06245 (cofilin), FGSG_01244 (pre-mRNA splicing factor), and FGSG_10791 (RNA helicase) were chosen as potential reference genes. Subsequently, qRT-PCR expression data for the reference genes were subjected to analysis by the software geNORM (http://medgen.ugent.be/~jvdesomp/genorm/) to check their expression stability [30]. All three selected reference genes yielded values for the gene-stability measure M below the threshold (M = 0.7) and were therefore used to derive the specific normalization factor (NF) for each RNA sample as reported [30]. Finally, for each gene-of-interest the mean R_0 _of the RNAs from treated and untreated mycelia were calculated and used to determine the log_2 _fold-change between the control and treatment.

## Results

### Design and validation of an 8 × 15 k microarray for *F. graminearum*

In this study, a new *Fusarium graminearum *microarray was designed for the 8 × 15 k Agilent multiplex format. The representation of most genes by a single oligonucleotide probe on the array demanded a thorough pre-selection. Potentially suitable 60 mer probes were determined by bioinformatics and then experimentally validated for hybridization-signal quality by a pre-selection microarray experiment. For 57% of the genes four to seven probes were assembled on the pre-selection array whereas 41% of the genes were represented by eight to nine probes. It was not possible to derive more than three probes for about 2% of the genes. For two of the annotated genes, FGSG_08274 and FGSG_12303, no specific oligonucleotide was identified, due to sequence duplication and repetitive sequence structure. Accordingly, 103,747 probes were assembled on a 2 × 105 k Agilent microarray that was hybridized to Cy3-labeled cRNA derived from a pool of RNAs from three cultures using different media. The second hybridization used labeled genomic DNA. Transcripts were detected for about 80% of the genes when applying a proprietary threshold adjustment (imaGenes, Berlin, Germany) to the processed signal data from the RNA hybridization (data not shown). Those 10,622 genes were considered to be expressed. A proprietary algorithm (imaGenes) selected the optimal oligonucleotides for the final 15 k array by using the results from the RNA hybridization for the expressed genes. For the 2725 genes that were not expressed, the signal data from the DNA hybridization were utilized. The final 15 k array design comprised 15,208 *F. graminearum*-specific and 536 additional oligonucleotides, including internal controls (Additional file 1). In total 11,476 genes and 10 ESTs that were derived from NIV-type TRI cluster genes were represented by one oligonucleotide, whereas for 1854 genes and 7 NIV-type TRI cluster ESTs two probes were chosen.

The general quality of the signals was assessed with regard to the background noise. For all hybridizations, the average percentage of spot data determined by the Feature Extraction Software to be positive and significantly above background was 96.1 ± 2.2%. The chosen one-color hybridization method is a rather recent development for the Agilent platform. Therefore, the performance of the array was evaluated by technical replications that used a single RNA sample from one of the untreated cultures for three hybridization experiments to determine variation arising during all stages of data generation. Pearson's correlation coefficients (R) of 0.992, 0.995, and 0.995 (*p *< 0.0001 each) indicated highly significant conformity. In comparison, the biological replicates of the untreated and the treated samples also showed strong correlation, with R = 0.939, 0.946, 0.952, and respectively 0.945, 0.958, 0.968 (*p *< 0.0001 each). The minor variations detected in these experiments demonstrated high experimental reproducibility.

### Gene expression in response to tebuconazole treatment

To validate the suitability of the microarray to suggest fungicide target(s) and mode(s) of action, *F. graminearum *strain PH1 was exposed to 5 ppm of tebuconazole in liquid culture for 12 h. This concentration was previously determined as sub-lethal but strongly inhibitory for fungal vegetative growth (about 95% on fungicide-amended agar-plates compared to untreated controls; data not shown).

Based on the processed, normalized gene signals the average log_2 _fold-change (log_2 _FC) between both experimental groups (tebuconazole treated vs. untreated control) was assessed for each gene (Additional file 3). Statistical analysis using the optimal discovery procedure (ODP) as implemented in the EDGE software resulted in a list ranking genes according to significance based on *q *values (Additional file 3). To identify genes that appeared differentially expressed after fungicide treatment, we applied two conditions, i.e. a cut-off for statistical significance (*q *< 0.04) and a cut-off for fold-changes (log_2 _FC ≥ 1 and ≤ -1). This procedure identified 596 genes with significantly increased and 462 genes with significantly decreased transcript abundances. The 15 most significantly differentially expressed genes with either an increase or a decrease in response to the fungicide are listed in Table 2. Among those with increased transcript levels were five genes (FGSG_04092, FGSG_09764, FGSG_03686, FGSG_13888, and FGSG_02771) that are putatively involved in sterol metabolic processes. Among the genes exhibiting decreased transcript levels were genes that affiliated with amino acid transport (FGSG_02499, FGSG_02950, and FGSG_09354) and amino acid metabolism (FGSG_10119, FGSG_02349, FGSG_00296, and FGSG_09647).

**Table 2:** Genes with most significantly altered transcript levels

**Gene ID **^**a**^	**Annotation **^**b**^	**log**_**2 **_**FC **^**c**^	***q ***^**d**^
	**15 most significantly increased**		
FGSG_04092	probable cytochrome P450 51 (eburicol 14 alpha-demethylase)	7.35	0.006
FGSG_02255	conserved hypothetical protein	6.57	0.006
FGSG_02748	related to endothelin-converting enzyme 1	2.80	0.012
FGSG_09764	related to phosphomevalonate kinase	3.33	0.012
FGSG_03686	probable cytochrome P450 (involved in C-22 denaturation of the ergosterol side-chain)	3.69	0.012
FGSG_04492	conserved hypothetical protein	4.61	0.012
FGSG_10490	conserved hypothetical protein	3.46	0.012
FGSG_10170	conserved hypothetical protein	1.80	0.012
FGSG_02721	conserved hypothetical protein	1.45	0.012
FGSG_13888	related to emopamil-binding protein	7.35	0.012
FGSG_03489	conserved hypothetical protein	4.35	0.012
FGSG_04874	conserved hypothetical protein	6.78	0.012
FGSG_05739	conserved hypothetical protein	6.94	0.013
FGSG_04034	conserved hypothetical protein	3.97	0.013
FGSG_02771	probable KES1 - involved in ergosterol biosynthesis	3.05	0.013
	**15 most significantly decreased**		
FGSG_02499	related to amino acid transport protein	-1.49	0.012
FGSG_06437	related to triacylglycerol lipase V precursor	-2.18	0.012
FGSG_13414	conserved hypothetical protein	-2.13	0.012
FGSG_00788	conserved hypothetical protein	-2.12	0.016
FGSG_10119	related to threonine dehydratase	-2.06	0.027
FGSG_02349	probable tyrosine-tRNA ligase	-2.33	0.029
FGSG_02950	related to neutral amino acid permease	-2.20	0.029
FGSG_09392	conserved hypothetical protein	-1.04	0.029
FGSG_09354	probable neutral amino acid permease	-2.16	0.029
FGSG_04215	related to monocarboxylate transporter 2	-3.20	0.032
FGSG_00296	probable ILV1 - anabolic serine and threonine dehydratase precursor	-1.94	0.033
FGSG_10809	conserved hypothetical protein	-1.75	0.033
FGSG_08394	conserved hypothetical protein	-1.38	0.033
FGSG_10626	related to phenazine biosynthesis protein phz	-2.16	0.033
FGSG_09647	probable histidinol-phosphate transaminase	-2.17	0.033

^a ^The given gene ID is the entry number of the *F. graminearum *genome annotation FG3. ^b ^Annotation as provided by the MIPS *Fusarium graminearum *genome database (FGDB). ^c ^The relative transcriptional differences between fungicide-treatment vs. untreated control given as log_2 _transformed fold-changes. ^d ^Statistical assessment of transcriptional differences by ODP. The *q *value is a measure of the false-positive discovery rate in a given set of genes.

Two functional annotation approaches, "The Functional Catalogue" (FunCat) and "GeneOntology" (GO), were used to determine whether distinct functional groups of proteins were overrepresented within the differentially expressed genes. Among the genes with increased transcript levels, these programs identified only few functional categories as significantly enriched. These analyses revealed "sterol biosynthetic process" and "tetracyclic and pentacyclic triterpenes (cholesterin, steroids and hopanoids) metabolism" as the most significantly enriched functional classes (Additional file 4). Furthermore, both analyses also identified isoprenoid metabolism and heme binding protein functions as significantly enriched. The two methods yielded distinct results for some additional categories. Whereas FunCat found protein degradation as significantly enriched, GO regarded proteins with hexosyl transferase activity and proteins involved in membrane processes and mycelium development as significantly enriched among the genes with increased transcript levels. The analyses of the genes with significantly decreased transcript levels retrieved a higher number of enriched categories. Both methods identified several central cellular processes, e.g. respiratory chain and energy generation and metabolism of amino acids and cofactors (Additional file 4).

### Analyses of selected gene subsets

Previous studies on azole response and/or resistance in human pathogens to azoles have implicated a general importance of genes encoding ergosterol biosynthesis proteins, ABC transporters and transcription factors. However, in plant pathogens corresponding information is scarce. Therefore, we examined these genes in more detail.

#### Ergosterol biosynthesis

Both functional enrichment analyses uncovered proteins belonging to sterol or steroid biosynthesis as the category exhibiting the most significant response to tebuconazole treatment. This was reflected by significantly increased transcript levels of most of the genes with homologs among the ergosterol biosynthesis genes from *S. cerevisiae *(Additional file 2).

**Figure 1:**
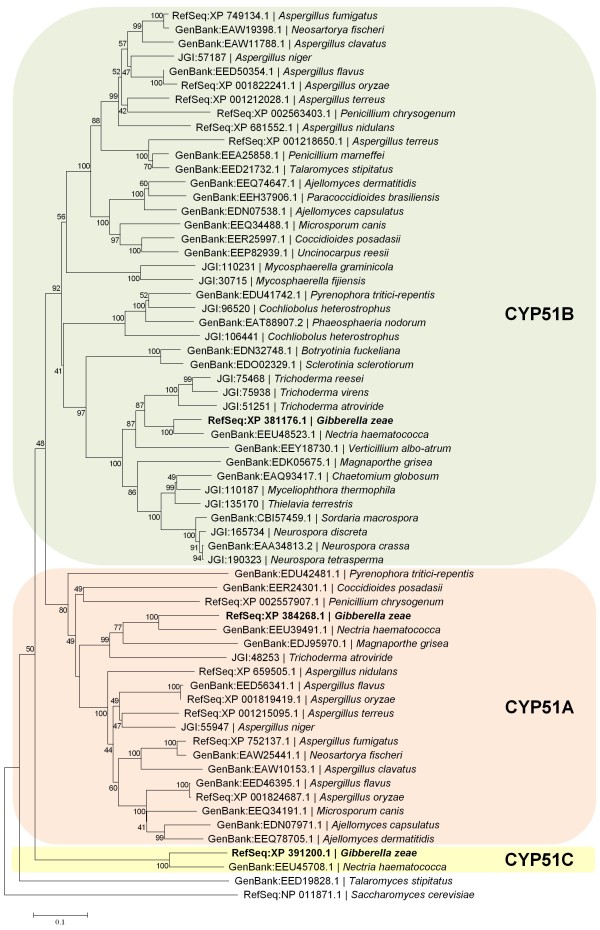
**Molecular phylogeny of CYP51 proteins of Pezizomycotina**. The depicted phylogram was obtained by Neighbor-Joining using MEGA4 software and reflects the relationships between 64 amino acid sequences of CYP51 (syn. ERG11) like proteins extracted from 39 annotated proteomes. Results from bootstrapping with 1000 replicates are indicated when higher than 30%.

In contrast to *S. cerevisiae*, certain *Erg *genes are represented by more than one copy in some Pezizomycotina, including *F. graminearum *[31]. Each of the *S. cerevisiae *genes *Erg3*, *Erg5, Erg6*, *Erg10*, and *Erg24 *corresponded to two genes in the genome of *F. graminearum*. Interestingly, three genes, i.e. FGSG_01000, FGSG_04092 and FGSG_11024, encoded ERG11 (syn. CYP51) the molecular target of azole action. Therefore, we analyzed the evolution of CYP51 in 38 species of the Pezizomycotina that have annotated proteomes. In total, 17 of the species had one and 14 had two *Cyp51 *genes. The genomes of five species, i.e. *F. graminearum *(teleomorph *Gibberella zeae*), *Fusarium solani *(teleomorph *Nectria haematococca*), *Aspergillus terreus*, *A. flavus, and A. oryzae*, harbor three putative *Cyp51 *genes. We created a molecular phylogeny to examine the relationships between the CYP51 proteins from Pezizomycotina and included *S. cerevisiae *as the outgroup (Figure 1). These analyses identified three clades for the CYP51 proteins of the Pezizomycotina, two of which had previously been termed CYP51A and CYP51B [32]. Species that had only one copy of CYP51 were exclusively found in clade B. In those species that encoded two or three CYP51 proteins one variant clustered in clade B, the other in clade A, except for *Cochliobolus heterostrophus *and *Talaromyces stipitatus*. The former possessed two CYP51B variants, whereas the latter showed a distinct CYP51 type that positioned at an unsupported branch. For species that encoded three CYP51 proteins in their genomes two patterns of clustering were observed for the third additional copy. In *Aspergillus *spp., the third CYP51 variants clustered either with clade A (*A. flavus *and *A. oryzae*) or with clade B (*A. terreus*). In contrast, the third CYP51 variants of *F. graminearum *(FGSG_11024) and *F. solani *formed a distinct and supported clade termed CYP51C.

Regarding the unique phylogenetic distribution of the three *Cyp51 *genes in *F. graminearum*, we analyzed whether they exhibited different transcriptional responses after azole treatment. qRT-PCR confirmed that the transcript levels of all three *Cyp51 *genes were significantly increased by the treatment (Table 3). The largest increase was observed for FGSG_04092 (= *Cyp51A*) that was also the most consistently differentially expressed gene of the microarray study (Table 2). The sigmoidal model applied to evaluate real-time kinetics allowed calculating the R_0 _value which reflects initial transcript abundance [33]. These comparisons revealed similar transcript abundances for FGSG_01000 (= *Cyp51B*) and FGSG_11024 (= *Cyp51C*) in untreated mycelia. Transcript abundances of these genes were about 10 times higher than that of FGSG_04092 (= *Cyp51A*) (Additional file 5). However, the response of the latter gene towards tebuconazole treatment appeared as most drastic, showing a 121-fold increase of transcript abundance.

**Table 3:** Comparison of differential gene expression as determined by microarray hybridization and qRT-PCR

**Gene ID **^**a**^	**Type **^**b**^	Microarray		qRT-PCR	
		**log**_**2 **_**FC **^**c**^	***q ***^**d**^	**log**_**2 **_**FC **^**c**^	***p ***^**e**^
**CYP51**					
FGSG_01000	CYP51B	2.96	0.0303	2.89	< 0.0001
FGSG_04092	CYP51A	7.35	0.0061	6.92	< 0.0001
FGSG_11024	CYP51C	2.06	0.0385	1.54	0.0001
**ABC transporters**					
FGSG_00046	MRP	3.00	0.0392	1.17	0.0231
FGSG_02786	MDR	2.16	0.0392	1.64	0.0036
FGSG_03032	MDR (half)	-1.29	0.0392	-0.99	0.0793
FGSG_03882	PDR	-3.65	0.0516	-3.27	0.0560
FGSG_05076	PDR (half)	1.94	0.0385	1.17	0.0249
FGSG_06771	MDR	2.02	0.0392	1.56	0.0017
FGSG_06881	MDR	1.38	0.0401	2.08	0.0303
FGSG_07325	MRP	1.57	0.0539	1.42	0.0033
FGSG_08308	MRP	4.07	0.0508	3.41	0.0310
FGSG_08309	PDR	-2.95	0.0509	-2.05	0.0686
FGSG_08312	PDR	2.67	0.0385	1.90	< 0.0001
FGSG_08373	n.d.	1.55	0.0395	1.32	0.0036
FGSG_08830	PDR	6.58	0.0523	3.78	0.0226
FGSG_10995	MRP	2.61	0.0497	1.30	0.0176
FGSG_11028	MRP	1.44	0.0502	-0.85	0.4518
FGSG_11988	MDR	-1.91	0.0509	-1.63	0.0264
**Transcription factors**					
FGSG_00069	Zn2Cys6	-2.14	0.0385	-1.81	0.0207
FGSG_01293	Zn2Cys6	2.08	0.0385	1.91	0.0010
FGSG_01341	C2H2	1.62	0.0357	1.92	0.0042
FGSG_01669	Zn2Cys6	3.86	0.0392	3.30	0.0002
FGSG_05949	WING	4.16	0.0385	3.96	< 0.0001
FGSG_06324	Zn2Cys6	1.64	0.0385	1.00	< 0.0001
FGSG_06810	Zn2Cys6	1.70	0.0387	1.35	0.0002
FGSG_09333	Zn2Cys6	-1.22	0.0385	-0.57	0.0121
FGSG_09349	Zn2Cys6	-2.16	0.0385	-0.98	0.0092
FGSG_10470	C2H2	4.74	0.0385	4.32	< 0.0001
FGSG_10914	Zn2Cys6	-1.03	0.0392	-1.14	0.0738
FGSG_11561	Zn2Cys6	1.48	0.0387	1.57	0.0162

^a ^The given gene ID is the entry number of the *F. graminearum *genome annotation FG3. ^b ^Classification to gene subfamilies. ^c ^The relative transcriptional differences between fungicide-treatment vs. untreated control given as log_2 _transformed fold-change as determined by microarray hybridization and by qRT-PCR. ^d ^Statistical assessment of transcriptional differences by ODP. The *q *value is a measure of the false-positive discovery rate in a given set of genes. ^e ^Statistical assessment of transcriptional differences by Student's t-test.

ALDp, adrenoleukodystrophy protein; CYP51, cytochrome P450 sterol 14α-demethylase; MDR, multi drug resistance proteins; MRP, multidrug resistance-associated proteins; PDR, pleiotropic drug resistance proteins; C2H2, Cys-Cys-His-His motif zinc finger proteins; WING, winged helix-turn-helix DNA-binding protein; Zn2Cys6, Zn(II)2-Cys6 binuclear cluster domain-type transcription factors; n.d. not defined.

##### ABC transporters

**Figure 2:**
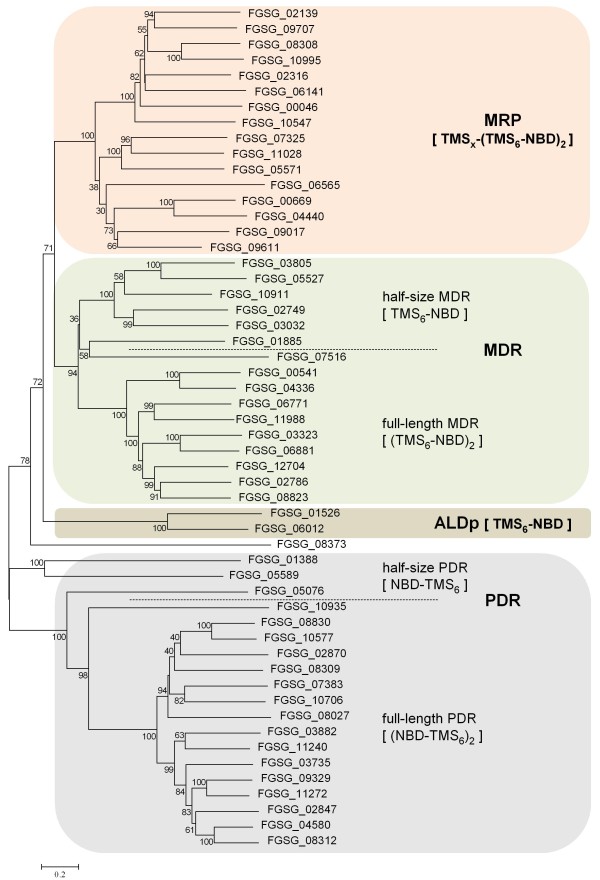
**Molecular phylogeny of *F. graminearum *ABC transporters**. The depicted phylogram was obtained by Neighbor-Joining using MEGA4 software and reflects the relationships between 54 amino acid sequences of putative ABC transporters that were extracted from the *F. graminearum *proteome. Results from bootstrapping with 1000 replicates are indicated when higher than 30%. Classification for subfamily uses a published nomenclature [26,27] and is indicated by colored boxes. ALDp, adrenoleukodystrophy protein; MDR, multi drug resistance proteins; MRP, multidrug resistance-associated proteins; PDR, pleiotropic drug resistance proteins; NBD, nucleotide-binding domain; TMS, transmembrane spanning domain.

Previous studies on azole responses in other fungi had indicated a contribution of ABC transporters to drug tolerance [34]. Using FunCat and Blast2GO annotations, 54 putative ABC transporter proteins were identified in the *F. graminearum *proteome. We constructed a phylogenetic tree to examine their relationships especially in the context of subfamilies known from ABC transporters [26,27] (Figure 2). Thereby, we classified multidrug resistance proteins (MDR), multidrug resistance-related proteins (MRP), pleiotropic drug resistance proteins (PDR), and adrenoleukodystrophy protein (ALDp)-type ABC transporters, which were represented by 16, 16, 19, and 2 members, respectively (Figure 2). One putative ABC transporter gene, FGSG_08373, did not group with any of these subfamilies established first in the Saccharomycotina. Its domain organization corresponded to MDR-type proteins, but it had highest similarity to PDR-type proteins from *C. albicans *and *S. cerevisiae*. Typically, ABC transporters comprise two transmembrane and two nucleotide-binding domains. However, for the MDR- and PDR-types, half-sized proteins with only one copy of the domains are also known. In *F. graminearum*, six half-size MDR and three PDR-proteins exist (Figure 2). Two sequences (FGSG_08027 and FGSG_09611) seemed to miss parts of the corresponding proteins putatively as a result of erroneous ORF predictions.

The microarray analyses indicated that transcript levels of 23 genes of the ABC superfamily showed more than twofold (log_2 _FC ≥ 1 and ≤ -1) changes after tebuconazole treatment, and seven that additionally met our significance threshold of *q *< 0.04 (Additional file 2). Of these, four belonged to the MDR-, two to the PDR-, and one to the MRP-type. One gene encoding a half-size MDR protein exhibited significantly reduced transcript levels. However, results from microarray analyses of gene families like the ABC transporters might be blurred by mispriming due to extended sequence similarities. Therefore, qRT-PCR was performed for 16 ABC transporter genes to evaluate the reliability of the microarray data (Table 3). For 15 of the selected genes the log_2 _FC values for tebuconazole treatment obtained by the microarray experiment were coherent with those of qRT-PCRs. For one gene, i.e. FGSG_11028, qRT-PCR did not confirm the microarray data. The relative transcript levels of different ABC transporter genes, as determined by qRT-PCR varied by up to a factor of 538 in untreated mycelia (see FGSG_02786 vs. FGSG_06771). In mycelia treated by tebuconazole, up to 778fold differences in transcript abundances were detected (FGSG_11028 vs. FGSG_06771) (Additional file 5).

##### Transcription factors

The microarray data were also analyzed to identify potential regulators involved in azole-induced transcriptional responses. According to the Fungal Transcription Factor Database, the *F. graminearum *proteome contained 660 putative transcription factors classified into 44 families (Additional file 2). The transcript levels of 22 of these genes were significantly increased, whereas 15 were decreased by the tebuconazole treatment (log_2 _FC ≥ 1 and ≤ -1; *q *< 0.04). BlastP analyses using the proteins of the increased genes as queries against the annotated proteomes of *S. cerevisiae *and *C. albicans *yielded hits with some proteins known to be involved in azole-stress responses in these yeasts (see Additional file 2). The proteins encoded by FGSG_06324, FGSG_10364, and FGSG_11561 resembled the sterol regulatory binding protein UPC2, which is a transcription factor controlling ergosterol biosynthetic and sterol uptake genes [35]. The protein encoded by FGSG_06810 was similar to the transcription factor TAC1, which is a regulator of ABC transporters [36]. Other up-regulated transcription factors appeared to be involved in rather general stress responses. FGSG_01341, FGSG_10470, and FGSG_13711 were similar to CRZ1, a transcription factor involved in calcineurin- and Ca^2+^/calmodulin-dependent signaling [37]. The proteins encoded by FGSG_09019, FGSG_01293, FGSG_01936, and FGSG_13828 are putative regulators of metabolic processes, which may be indirectly compromised by fungicide treatment. These genes exhibit similarities to yeast's transcription factors involved in phosphate metabolism, carbohydrate metabolism, and fatty acid degradation (see Additional file 2).

Among the 13 putative transcription factors, which exhibited significantly reduced transcript levels after tebuconazole treatment, FGSG_09333 showed similarity to RDR1 of *S. cerevisiae *[38]. RDR1 is a repressor of the ABC transporter gene *Pdr5 *encoding an efflux pump which is important for multidrug resistance in yeast. FGSG_12970 was similar to the transcription factor RIM101 described as a positive [39] and negative [40] regulator protein contributing to cell wall assembly and altered lipid asymmetry signaling in yeast. Due to their homologies to the yeast proteins DAL81 and ARG81, the *F. graminearum *proteins encoded by FGSG_10914 and FGSG_00144 might be involved in regulation of catabolic processes.

qRT-PCR analyses were performed for 12 genes encoding transcription factors and showed high congruency to the respective array data (Table 3). The qRT-PCR data suggested considerable intergenic differences of absolute transcript abundances (Additional file 5).

### Comparison of transcriptomic responses to different types of stress

**Figure 3:**
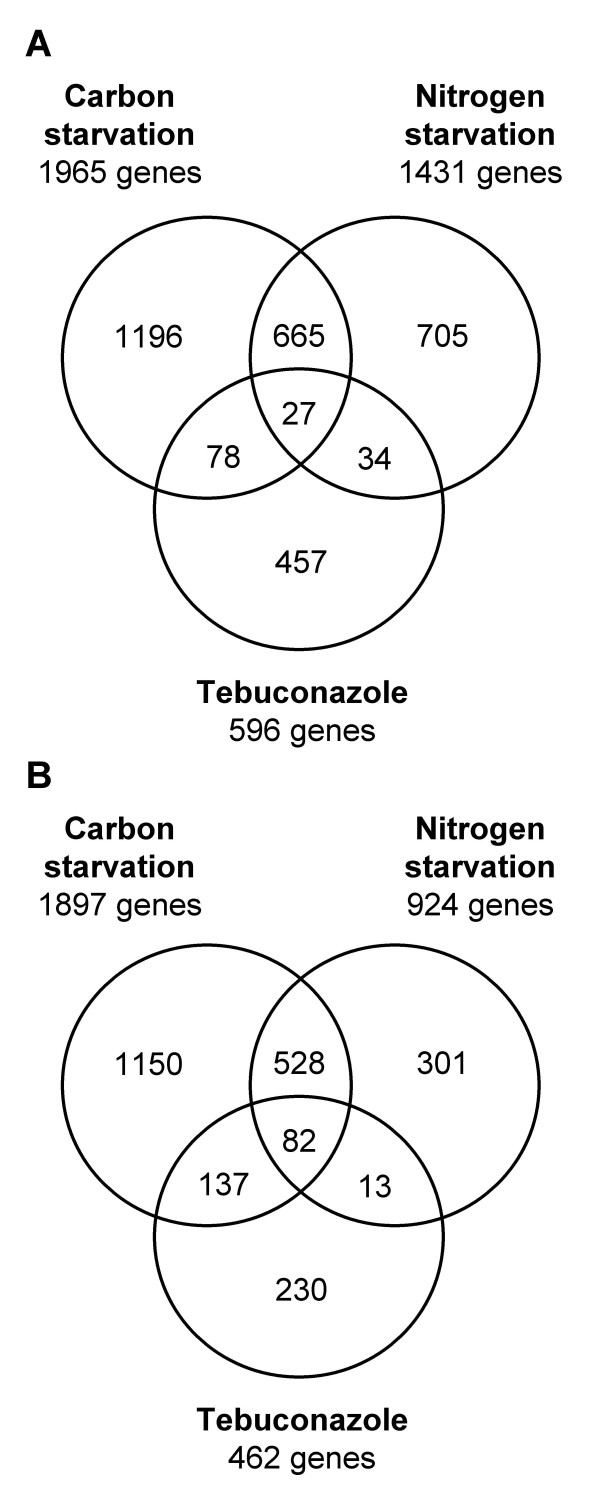
**Comparison of differentially expressed genes exposed to different stress conditions**. Venn diagrams illustrate results from comparative analyses of gene sets found differentially expressed by azole treatment, carbon, or nitrogen starvation [10]. **A**. Comparisons of gene sets showing significantly increased transcript levels. Nine of 596 genes found in the current tebuconazole study did not match a probe set on the Affymetrix GeneChip. **B**. Comparisons of genes sets showing significantly decreased transcript levels. Three out of 462 genes did not match a probe set on the Affymetrix GeneChip.

We compared our microarray data to those of a study that was previously published for *F. graminearum *[10] to assess how many of the genes responding to tebuconazole would also respond to other types of stress. We applied the same statistical procedures described above to the published microarray data that examined two nutritional stress conditions, i.e. carbon and nitrogen starvation. Based on the *q *value plots obtained by the EDGE software we used *q *< 0.01 as the cut-off for statistical significance for these data sets. In addition, transcript levels in the published data needed to be changed at least twofold (log_2 _FC ≥ 1 and ≤ -1) by the respective treatment to be considered significant. Resulting gene sets were used for subtractions (Figure 3). Transcript abundances from the majority, i.e. 457 (76.7%) of the above described 596 genes were specifically increased after the fungicide treatment whereas 139 (23.3%) genes responded also in at least one of the other two stress conditions (Figure 3 A; Additional file 6). Some of those belonged to one of the three groups of proteins discussed above. FGSG_02346 (homolog of the *S. cerevisiae *gene *Erg24*), FGSG_09381 (homolog of *S.c. Erg9*) and FGSG_10424 (homolog of *S.c. Erg19*) belonged to the ergosterol biosynthesis pathway, whereas for ABC transporters and transcription factors we correspondingly identified three genes (FGSG_02786, FGSG_06771, FGSG_08312), and five genes (FGSG_01293, FGSG_01307, FGSG_01936, FGSG_06324, FGSG_10639), respectively. However, functional enrichment analysis with the remaining 457 genes whose transcripts were specifically increased by tebuconazole still indicated a statistically significant enrichment of genes involved in steroid metabolism (data not shown).

Comparison of the three stress conditions with respect to genes showing reduced transcript levels indicated considerably overlapping responses (Figure 3B). Of the 462 genes transcriptionally decreased after tebuconazole treatment 232 (50.2%) were also decreased in at least one of the other two conditions. This included the genes FGSG_00069, FGSG_09177, FGSG_09333, FGSG_09349, FGSG_09852, FGSG_12970, FGSG_13172, all of which encode transcription factors. Importantly, none of the genes encoding enzymes of ergosterol biosynthesis or ABC transporters exhibited decreased transcript levels by the starvation stresses. Additional BlastP analyses focussing on those genes that were indicated to be specifically transcriptionally increased or decreased by the tebuconazole treatment revealed 42, respectively 11 genes that appeared to be unique to *F. graminearum *as they had no similarity at e <10^-10 ^to any other sequence in the current database (Additional file 6). Seventeen additional genes showing transcript levels specifically increased by tebuconazole found similar genes only in other *Fusarium *species (Additional file 6). The same applied to nine additional of the down-regulated genes.

## Discussion

In this study, we developed and validated a new 8 × 15 k Agilent microarray, and employed it to analyze gene expression in *F. graminearum *after treatment with tebuconazole. Our results demonstrate that this multiplex microarray is an effective and versatile tool to detect transcriptome responses at high sensitivity.

Due to its scientific and economic relevance, microarray technology has rapidly evolved into different platforms using short or long oligonucleotides provided by several commercial manufacturers, such as Affymetrix and Agilent Technologies. As studies using different microarray platforms indicated an overall good comparability [41], the choice of a platform is mainly governed by practical and cost considerations. Agilent's inkjet-like printing technology provides high flexibility for microarray design and allows convenient optimization in follow-up versions. In addition, multiplex formats were developed that carry several microarrays on a single slide, allowing for cost-effective transcription profiling. The chosen Agilent 8 × 15 k format is fully sufficient for covering the entire genome of *F. graminearum*, thus permitting to simultaneously perform eight independent expression profiling experiments. The employment of the one-color labeling technique recently introduced for the Agilent platform facilitates comparisons across microarrays and between groups of samples without compromising the quality of results [42].

To address the reliability of the results obtained by the new microarray, we selected 31 genes for determining their transcript levels by an independent experimental approach, i.e. qRT-PCR. For most of these genes, the log_2 _FC values from the qRT-PCRs were very close to those of the microarray experiments. The comparison of the entire data sets reveals a highly positive correlation (R = 0.95) which corroborates the results of a previous report that also examined these two experimental approaches [43].

The new 8 × 15 k Agilent microarray was used to analyze gene expression patterns in *F. graminearum *treated with tebuconazole. We assess the results in the context of microarray experiments that were performed earlier. On the one hand, results that are supported by those previously observed (e.g. in *S. cerevisiae*) highlight the capability of the new microarray to gain information relevant for better understanding the regulatory networks mediating azole responses. This will be especially helpful for studying novel fungicides whose mode-of-action should not only be analyzed in a model organism like *S. cerevisiae *but ideally also in the targeted pathogens, e.g. *F. graminearum*. Consistent with studies in other fungi [44-47] we found that the transcript levels of most of the genes encoding proteins involved in ergosterol-biosynthesis were increased by azole treatment. Furthermore, our functional enrichment analyses using GeneOntology and FunCat demonstrated that the ergosterol pathway ranks at the top among all functional categories containing genes with significantly enhanced transcript abundances. Interestingly, we found that within this category FGSG_04092 (= *Cyp51A*), which is one of the three genes encoding CYP51 in *F. graminearum*, exhibited the most significantly increased transcript levels of the entire study. In *S. cerevisiae *binding of azoles to CYP51 leads to ergosterol depletion, accumulation of a toxic aberrant sterol and compromised membrane rigidity [48]. It is thus possible that a feed-back loop connecting transcriptional regulation of ergosterol biosynthesis and sterol levels exists in *F. graminearum*, as previously reported for *S. cerevisiae *[49]. In yeast this link is mediated by UPC2 and ECM11 which regulate transcription of sterol biosynthetic genes by binding to a sterol regulatory element (SRE) in their promotors [35].

The fact that our microarray study uncovered the transcript levels of genes in the ergosterol biosynthesis pathway as highly significantly increased by tebuconazole treatment demonstrates its utility for fungicide research. The data presented here clearly indicate that microarray analysis can contribute to identify unknown mode-of-actions, as has previously been shown for the fungicide ciclopirox olamine in *Candida albicans *[50]. Sixty percent of the up-regulated genes were found to encode proteins for iron uptake and metabolism suggesting that treated cells suffered from iron limitation. This was supported by physiological experiments showing that addition of Fe^2+ ^or Fe^3+ ^diminished the detrimental effect of ciclopirox olamine on germ tube formation.

Comparison of our results with published microarray data indicates that about one quarter of the genes up-regulated by tebuconazole and almost half of the down-regulated genes correspondingly responded when *F. graminearum *was exposed to two unrelated starvation stress conditions. This suggests that many of the genes exhibit a rather unspecific stress response, which may in part originate from defective nutrient supply resulting from the azole-mediated membrane perturbations. On the other hand, the majority of the genes with enhanced transcript abundances responded specifically to fungicide treatment, which is underlined by the fact that all *Cyp51 *variants only responded in our azole treatment study. Thus, the novel microarray is an excellent tool supporting identification of target genes. Microarray data of course need to be confirmed in subsequent studies, e.g. by targeted gene deletion or RNAi experiments, to address the function of individual genes to attenuate fungicide impact and the mechanisms by which this may be achieved.

Currently it is uncertain whether the CYP51A, B, and C proteins have specific functions in *F. graminearum*. In *Aspergillus fumigatus *transformants carrying individual deletions of *Cyp51A *and *Cyp51B *remained viable whereas the gene family as a whole was essential [51]. However, in this human pathogen up to now only point mutations in *Cyp51A *but not in *Cyp51B *were discovered in clinical isolates and resistant strains generated *in vitro *[52-54]. This suggests that the functions of these proteins may not completely overlap.

Also ABC transporters were reported to be involved in azole stress responses and in the development of azole tolerance in several fungi as some of them mediate active efflux of fungicides and other xenobiotics [34]. The subfamily most closely associated with drug resistance in the Saccharomycotina is PDR which have up to 10 members in yeasts [55]. In contrast, the genome of *F. graminearum *harbors 19 PDR-type ABC transporters. An evolutionary expansion of the PDR subfamily was previously noticed for Pezizomycotina [56]. In addition, the numbers of genes encoding MRP and MDR transporters (16 each) are clearly increased in *F. graminearum *as compared to *S. cerevisiae *(4 and 7, respectively).

Our microarray data provide valuable insight into the transcriptional responses of ABC transporter genes from *F. graminearum*, suggesting that several of them may attenuate the effects of tebuconazole action. Similarly, microarrays analyzing azole responses of *C. albican*s wild type strains showed that the transcript levels of the ABC transporter genes *Cdr1 *and *Cdr2 *were increased [46]. Both genes were also up-regulated in *in vitro *azole-adapted strains and in clinically resistant isolates [57,58]. Gene deletions in an azole-resistant isolate of *C. albicans *indicated that CDR1 is essential to mediate azole resistance whereas CDR2 seems to be less important [59]. However, heterologous overexpression of both *Cdr1 *or *Cdr2 *conferred increased azole tolerance to *S. cerevisiae *[60]. Another well-characterized ABC transporter is PDR5 of *S. cerevisiae*, which is exporting a wide range of xenobiotics, including azoles [60,61]. Additional reports underline the involvement of ABC transporters in contributing to fungicide resistance also in plant pathogenic Pezizomycotina [62,63]. Since most of this research has focused on PDR-type transporters, it will be interesting to analyze also the contribution of azole-responsive MDR- and MRP-type proteins in the future.

Transcription factors have been identified as additional elements in conferring fungicide resistance [64,65]. Since the corresponding transcriptional networks vary to some degree when comparing species in the Saccharomycotina [64,66] it is important to extend such analyses to Pezizomycotina, including toxigenic plant pathogens like *F. graminearum*. Among the genes with significantly increased transcript levels, we detected several similarities with genes encoding the transcriptional regulators *Upc2 *and *Tac1 *known to be involved in azole responses in *S. cerevisiae *and/or *C. albicans*. Also in *C. albicans *transcript levels of *Upc2 *and *Tac1 *increased in response to azole-stress [36,67]. In both yeasts, UPC2 homologs are activators of genes encoding proteins for ergosterol-biosynthesis and sterol uptake [35,67-69]. In *C. albicans *a disruption of *Upc2 *induced hypersusceptibility and its overexpression increased azole resistance [70]. A G648D exchange in UPC2 created an allele that constitutively up-regulated *Erg11 *expression and thereby improved azole resistance. Similarly, *Tac1 *in *C. albicans *and *Pdr1 *in *S. cerevisiae *regulate the PDR-type ABC transporter genes *Cdr1, Cdr2*, and *Pdr5*, respectively [36,71]. In these two yeasts transcription factors mediate the response to azoles by regulating the expression not only of genes for ergosterol biosynthesis but also of efflux transporters. Other putative regulators with significantly increased transcript levels were similar to CRZ1 from *S. cerevisiae *and *C. albicans*, a transcription factor that is involved in regulating cell wall integrity [37]. This may reflect a more general response of the fungus in surviving the fungicide treatment. Apart from putative transcription factors with similarity to proteins reported to coordinate azole stress responses in *S. cerevisiae *and *C. albicans *we found additional azole-responsive transcription factors in *F. graminearum*. The response of these factors may reflect differences in transcriptional regulation of azole response that are either specific for *F. graminearum *or higher taxonomic levels.

## Conclusions

This study introduces a novel *F. graminearum *microarray that is flexible and cost effective due to its multiplex format. Gene expression analyses investigating the tebuconazole response indicated that this array when analyzed with proper bioinformatic tools will be very helpful in fungicide research, e.g. in elucidation of mechanisms of fungicide resistance or mode-of-action, and beyond. Detailed analyses of genes encoding ergosterol biosynthesis proteins, ABC transporters, and transcription factors established a base for functional studies of the *F. graminearum *regulatory network responding to azole exposure.

## Authors' contributions

RB contributed to conception of the study, collected and provided samples for microarray hybridizations, performed data analysis, initiated qRT-PCRs, and drafted the manuscript. FW conducted several qRT-PCR assays. HD was an advisor of the work and contributed to the manuscript. SW conceived and coordinated the project, and wrote the manuscript.

## Supplementary Material

Additional file 1 **Design of the 8 × 15 k *F. graminearum *microarray**. This file includes information about gene-specific oligonucleotides and additional features assembled on the 15 k microarray. In addition, the list of the corresponding transcript and gene IDs is provided for proper assignment of array oligonucleotides and data to a specific *F. graminearum *gene. Click here for file

Additional file 2 **Supplementary information about selected gene subsets**. This file provides information and results about the analyzed genes encoding proteins for ergosterol biosynthesis, ABC transporters, and transcription factors. Beside statistical data of microarray experiments (log_2_FC, *q *value, rank), information include BlastP results and gene descriptions for homologs in *S. cere*visiae and *C. albicans *obtained from the databases SGD and CGD, and transcription factor classification data from FTFD. Click here for file

Additional file 3 **Statistical results of microarray hybridizations**. This file includes information about statistical analyses with the software EDGE. Results shown include processed and normalized signal data used as input data and provides the original output data (Target ID, rank, *q *value, graphics). In addition, quantitative data of the fold-change (FC, log_2 _differences) are shown. Moreover, the file provides a complete list of genes with significantly increased resp. decreased transcript abundances. Click here for file

Additional file 4 **Functional enrichment analyses**. This file includes results of analyses using the functional annotation databases GeneOntology and FunCat. Information is provided for protein functions determined as significantly enriched in sets of *F. graminearum *genes showing significantly altered transcript levels after tebuconazole treatment. Results are presented separately for the two databases as well as with respect to increased and decreased transcript levels. Click here for file

Additional file 5 **Comparison of transcript levels of selected genes as determined by qRT-PCR**. The initial fluorescence R_0 _is a relative measure for the abundance of transcripts. R_0 _was calculated by application of sigmoidal curve-fitting. Click here for file

Additional file 6 **Supplementary data to Figure 3**. This file provides detailed gene lists, the Venn diagrams in Figure 3 were based on. Click here for file
