# Supplementary material for: Development of a novel multiplex DNA microarray for Fusarium graminearum and analysis of azole fungicide responses
Source: BMC Genomics. 2011 Jan 21;12:52. doi: 10.1186/1471-2164-12-52 (PMC3037902; doi:10.1186/1471-2164-12-52)
Supplement: Additional file 5 — Comparison of transcript levels of selected genes as determined by qRT-PCR. The initial fluorescence R0 is a relative measure for the abundance of transcripts. R0 was calculated by application of sigmoidal curve-fitting. [file 1471-2164-12-52-S5.PPT]

## Slide 1
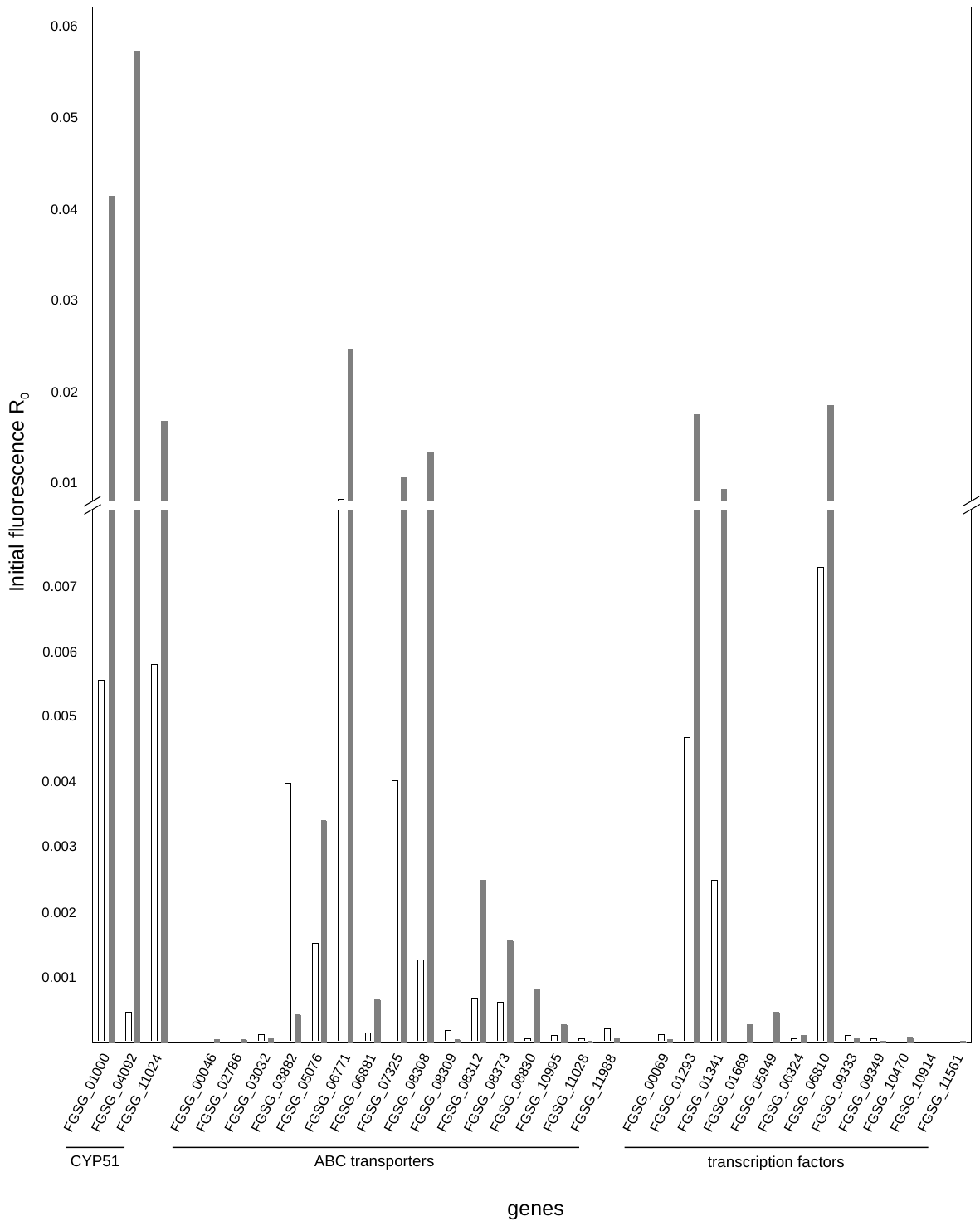

0.06
0.05
0.04
0.03
0.02
Initial fluorescence R0
0.01
0.007
0.006
0.005
0.004
0.003
0.002
0.001
FGSG_01669
FGSG_01341
FGSG_03882
FGSG_05076
FGSG_06881
FGSG_08308
FGSG_08309
FGSG_10995
FGSG_11988
FGSG_09333
FGSG_10914
FGSG_01000
FGSG_04092
FGSG_11024
FGSG_00046
FGSG_02786
FGSG_03032
FGSG_06771
FGSG_07325
FGSG_08312
FGSG_08373
FGSG_08830
FGSG_11028
FGSG_00069
FGSG_01293
FGSG_05949
FGSG_06324
FGSG_06810
FGSG_09349
FGSG_10470
FGSG_11561
CYP51
ABC transporters
transcription factors
genes
